# Viral Outbreak in Corals Associated with an *In Situ* Bleaching Event: Atypical Herpes-Like Viruses and a New Megavirus Infecting *Symbiodinium*

**DOI:** 10.3389/fmicb.2016.00127

**Published:** 2016-02-22

**Authors:** Adrienne M. S. Correa, Tracy D. Ainsworth, Stephanie M. Rosales, Andrew R. Thurber, Christopher R. Butler, Rebecca L. Vega Thurber

**Affiliations:** ^1^Department of Microbiology, Oregon State UniversityCorvallis, OR, USA; ^2^BioSciences at Rice, Rice UniversityHouston, TX, USA; ^3^ARC Centre of Excellence for Coral Reef Studies, James Cook UniversityTownsville, QLD, Australia; ^4^College of Earth, Ocean, and Atmospheric Sciences, Oregon State UniversityCorvallis, OR, USA; ^5^Department of Viticulture and Enology, University of California at DavisDavis, CA, USA

**Keywords:** virome, tropical coral reef, virus-like particle (VLP), herpesvirus, megavirus, nucleocytoplasmic large DNA virus (NCLDV)

## Abstract

Previous studies of coral viruses have employed either microscopy or metagenomics, but few have attempted to comprehensively link the presence of a virus-like particle (VLP) to a genomic sequence. We conducted transmission electron microscopy imaging and virome analysis in tandem to characterize the most conspicuous viral types found within the dominant Pacific reef-building coral genus *Acropora*. Collections for this study inadvertently captured what we interpret as a natural outbreak of viral infection driven by aerial exposure of the reef flat coincident with heavy rainfall and concomitant mass bleaching. All experimental corals in this study had high titers of viral particles. Three of the dominant VLPs identified were observed in all tissue layers and budding out from the epidermis, including viruses that were ∼70, ∼120, and ∼150 nm in diameter; these VLPs all contained electron dense cores. These morphological traits are reminiscent of retroviruses, herpesviruses, and nucleocytoplasmic large DNA viruses (NCLDVs), respectively. Some 300–500 nm megavirus-like VLPs also were observed within and associated with dinoflagellate algal endosymbiont (*Symbiodinium*) cells. Abundant sequence similarities to a gammaretrovirus, herpesviruses, and members of the NCLDVs, based on a virome generated from five *Acropora aspera* colonies, corroborated these morphology-based identifications. Additionally sequence similarities to two diagnostic genes, a MutS and (based on re-annotation of sequences from another study) a DNA polymerase B gene, most closely resembled *Pyramimonas orientalis* virus, demonstrating the association of a cosmopolitan megavirus with *Symbiodinium*. We also identified several other virus-like particles in host tissues, along with sequences phylogenetically similar to circoviruses, phages, and filamentous viruses. This study suggests that viral outbreaks may be a common but previously undocumented component of natural bleaching events, particularly following repeated episodes of multiple environmental stressors.

## Introduction

Viruses (phages and eukaryotic viruses) are abundant and diverse residents of stony coral colonies (reviewed in Vega Thurber and Correa, 2011). These viruses likely play multiple, parasitic and commensal roles in the health of coral reefs (e.g., Wilson et al., 2005; van Oppen et al., 2009; Rosenberg and Zilber-Rosenberg, 2014; Bettarel et al., 2015; Weynberg et al., 2015). Research interrogating the impact of viruses on colony fitness and survival under different environmental contexts is of particular importance, given anthropogenic climate forcing and other impacts (van Oppen et al., 2009). For example, abiotic conditions that stress coral colonies, such as elevated seawater temperatures or UV exposure, may trigger viral infections that contribute to coral bleaching and disease (Vega Thurber and Correa, 2011; Wilson, 2011; Lawrence et al., 2015). Identifying potential mechanisms of coral reef decline is increasingly important given accelerations in this process during recent decades (e.g., Gardner et al., 2003; De’Ath et al., 2012), and the current global mass bleaching event^1^.

Although the field of coral virology remains in its infancy, several groups have applied microscopy or genomics to examine the diversity and roles of viruses in coral holobionts. Microscopy studies have presented evidence that virus-like particles (VLPs) are present in all tissue layers of apparently healthy and diseased corals: the gastrodermis, mesoglea, and epidermis, as well as in the coral surface microlayer (CSM; e.g., Patten et al., 2008; Leruste et al., 2012; Bettarel et al., 2013; Nguyen-Kim et al., 2014; Pollock et al., 2014). The physical structure of VLPs also has been examined within cultured *Symbiodinium* (Wilson et al., 2001; Lohr et al., 2007; Lawrence et al., 2014). Some of these observed VLPs likely represent particles produced during the lytic replication phase of previously latent or endogenous infections of the coral animal, its dinoflagellate algae, or its microbiota (Patten et al., 2008; Wilson, 2011). Davy and Patten (2007), for example, were able to distinguish 17 sub-groups of VLPs associated with the CSM of four species of Australian corals based on morphological similarities. The role of each of these groups of viruses is uncertain, especially considering that the density of some VLPs within the CSM is relatively low. However, in other cases, transmission electron microscopy has revealed structures within corals that are highly indicative of massive viral infection (e.g., crystalline arrays, viral factories; Lawrence et al., 2014).

Yet standalone transmission electron microscopy (TEM) images can pose interpretive challenges. A set of TEM images may contain VLPs that present only some of the diagnostic morphological characteristics of a viral group, or characteristics that appear representative of many described viral groups (e.g., **Figure 3** in Vega Thurber and Correa, 2011). Further, since viral Families can encompass a range of capsid sizes and shapes and may overlap in these characteristics, microscopy-based studies may not fully resolve a group of VLPs. VLPs reminiscent of a large group of phylogenetically related viruses, the nucleocytoplasmic large DNA viruses (NCLDVs), exemplify this issue (e.g., Patten et al., 2008). The NCLDV group includes the *Phycodnaviridae*, *Iridoviridae*, *Poxviridae*, *Mimiviridae*, and *Ascoviridae*, as well as the recently described giant viruses, marseillevirus and lausannevirus (Iyer et al., 2006; Yamada, 2011; Yutin and Koonin, 2012). VLPs that are within the cytoplasm, larger than 120 nm, and generally icosahedral in shape are often interpreted as NCLDV-like but several exceptions to this rule remain, such as the poxviruses, and pandoravirus, which are NCLDVs that exhibit very different morphological characteristics.

Genomic and proteomic-based studies have identified patterns in the diversity and abundance of genomic sequences similar to described viruses within healthy and diseased tropical corals (Wegley et al., 2007; Marhaver et al., 2008; Vega Thurber et al., 2008; Littman et al., 2011; Weynberg et al., 2014; Wood-Charlson et al., 2015) and cultured *Symbiodinium* (Wilson et al., 2005; Claverie et al., 2009; Weston et al., 2012; Correa et al., 2013; Lawrence et al., 2014; Nguyen-Kim et al., 2014; Soffer et al., 2014a,b), as well as in cold water corals (Maier et al., 2011; Rosario et al., 2015). For example, a strong correlation between specific viral markers in bleached, diseased, and healthy *Orbicella* corals was used to establish a role for small circular ssDNA viruses (SCSDVs) in white plague disease (Soffer et al., 2014a). Although this work was somewhat substantiated by a TEM study on another coral white disease (Pollock et al., 2014), viral metagenome studies often contain sequence similarities to many viral groups, most of which have not been corroborated by microscopy-based studies. For example, numerous studies have found sequences similar to mimiviruses and baculoviruses, and yet no TEM study has confirmed these annotations (Claverie et al., 2009; Sharma et al., 2014; Wood-Charlson et al., 2015). This is likely due to several issues. A single sequence read or contig may have significant similarity to multiple viral groups because many related viruses share some gene homology. Alternatively, reads or contigs may have only a few sequence similarities with relatively high associated *e*-values. It can also be difficult to rule out contamination as a source of error for sequence similarities represented by few reads within a metagenome, or for reads similar to cosmopolitan or host-associated viral remnants (e.g., retrotransposons, retroelements). Thus, ambiguity remains when metagenomics is the sole approach applied to characterize the viral consortia associated with corals.

A comparative analysis of several metagenome studies recently addressed some of these challenges and cataloged a cosmopolitan set of viruses in corals and their symbionts (Wood-Charlson et al., 2015). This meta-analysis showed that, based on presence in >90% of 35 surveyed metagenomes, coral holobionts contain signatures of nine major Families in the dsDNA Group I viral lineages. These families include all of the major Caudovirales (*Sipho-*, *Podo-*, and *Myoviridae*) and many eukaryotic viruses. Within the eukaryotic viruses, the Herpesvirales Order, as well as five Families (*Phycodnaviridae*, *Iridoviridae*, *Poxviridae*, *Mimiviridae*, and *Ascoviridae*) within the NCLDVs are members of this 90% carriage cosmopolitan virome. Genomic signatures from some ssDNA (e.g., *Circoviridae*) and ssRNA (e.g., *Retroviridae* and *Caulimoviridae*) viral lineages are also well represented, but fall below a 90% threshold, likely due to biological and technical differences among studies (for discussion, see Wood-Charlson et al., 2015).

Regardless of the approach applied, a body of evidence indicates that herpes-like viruses and one or more NCLDVs associate with coral holobionts. Yet neither microscopy nor metagenomics alone has fully resolved the identity of either viral group within corals. Thus, this study sought finer taxonomic resolution for one or more groups by characterizing the viruses associated with fragments of dominant reef-building Pacific acroporid corals using morphological and sequence-based approaches in tandem. The aims of this work were to: (1) identify and compare specific VLPs in coral tissues; and (2) improve delineation of the core coral virome through the use of visual descriptors of viral taxonomy in conjunction with metagenomics analysis.

## Materials and Methods

### Overview of Environmental Setting, Experimental Setup, and Design

**FIGURE 1:**
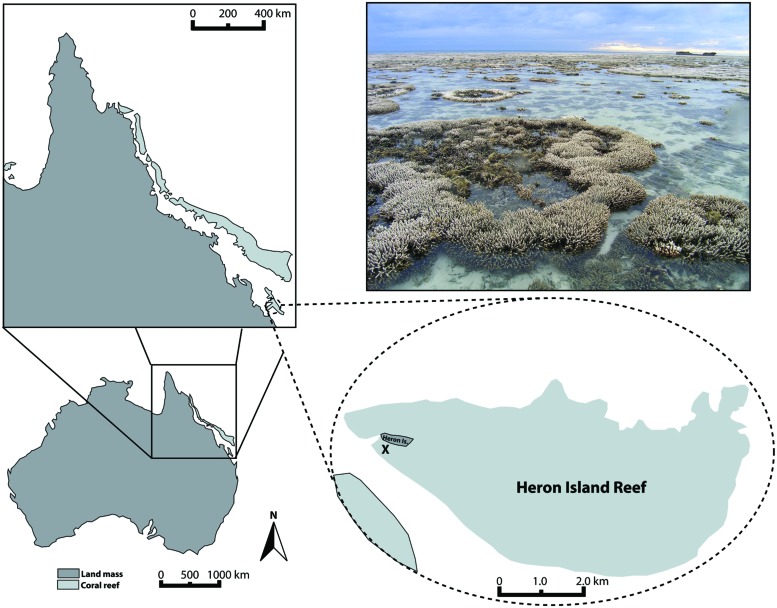
**Map of Heron Island tidal flat (Great Barrier Reef, Australia) indicating the location from which experimental coral colonies were collected (X)**. Photograph of tidal flat exemplifies the partial aerial exposure and associated patchy bleaching that many corals experienced in March 2011, prior to and in conjunction with the collection of experimental coral colonies.

**FIGURE 2:**
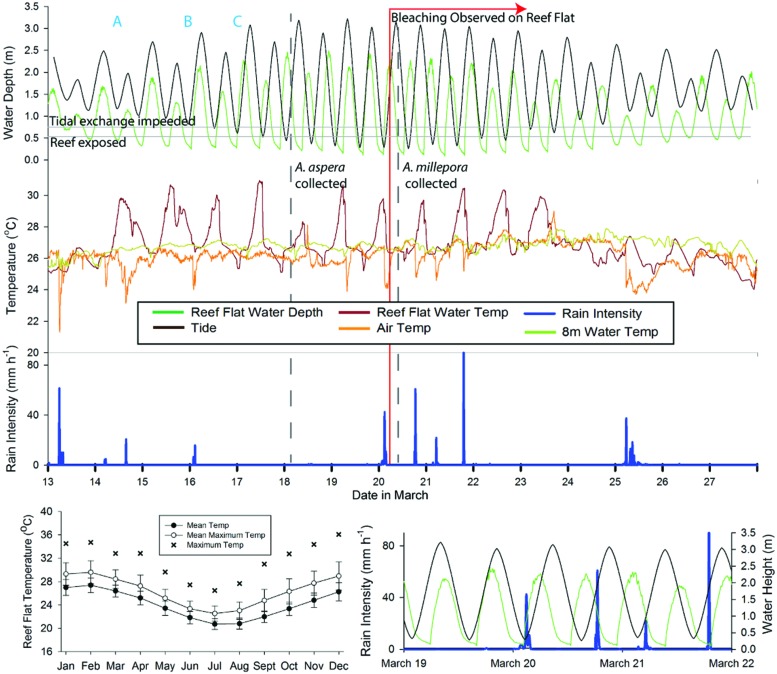
**Summary time line of conditions at the time of collection and observed bleaching at Heron Island**. Upper composite graph generally indicates (from top to bottom) tide, temperature, and rain intensity. Tide is based on tide tables from the region (black line), with reef flat water depth (green line) as measured at the 1.1 m mooring present on the reef flat. Temperature is depicted as reef flat water depth (dark red line, measured at the 1.1 m water depth mooring), 8 m temperature (light green line, measured at the 7.9 m mooring), and air temperature (orange line). Key aspects of the temperature and tidal cycle (time points A, B and C in upper composite graph), the timing of coral collections (vertical dashed gray lines) and the authors’ first observation of mass bleaching on the reef flat (vertical red line) are also indicated. Lower right graph is a subset of information from the upper composite graph, highlighting the overlap between low tides on the reef flat (green line, measured at the 1.1 m water depth mooring), the tidal height (black line), and rainfall intensity (blue line). Air temperature and rain intensity were obtained from the local weather station on Heron Island. Lower left graph summarizes the mean temperature (±1 SD) and mean maximum temperature (±1 SD) and maximum temperature recorded at the reef flat (1.1 m water depth mooring) for the years 2008–2015. This figure is based on data provided by the Australian Institute of Marine Science.

The experiments reported here were conducted using acroporid colonies (*N* = 5 for *A. aspera*; 4 for *A. millepora*) collected from the tidal reef flat off of Heron Island, Queensland, Australia (23°26′39.63′′S, 151°54′46.70′′E, **Figure 1**) in March of 2011. To evaluate the ambient environmental conditions at the study site, we used the Integrated Marine Observing System^2^ run by the Australian Institute of Marine Science to characterize the following parameters: rain intensity and timing, air temperature, water temperature (on the reef flat and at ∼8 m depth on the reef slope), and water height variations. Prior to and during our study, this reef flat experienced a period of low tides that caused repeated aerial exposure of reef flat colonies (e.g., **Figure 1** photograph) and increased residence time of water on the reef flat. Heavy rainfall was coincident with some low tides and aerial exposure of colonies. The temperature range experienced on the reef flat (measured at 1.1 m depth) was neither unique for the season nor as extreme as previous months, however (**Figure 2**, *Environmental Setting* in the Supplementary Material).

Corals were collected for the *A. aspera* experiment on March 18, 2011 (**Figure 2**). The experiment was initiated on March 19, 2011 and ran for 6 days. Corals were collected for the *A. millepora* experiment on March 20, 2011. The *A. millepora* experiment was initiated on March 22, 2011 and ran for ∼4.75 days. We hypothesized that aerial exposure and rainfall stressors prior to collection might have triggered bleaching in our acroporid colonies, and primed them for viral production. Therefore, we placed our coral colonies in flow-through (∼3 L min^–1^) seawater tanks and characterized their health states for at least 24 h prior to initiating the experimental periods. All colonies collected for use in this study exhibited normal pigmentation and were “apparently healthy” from the time of collection through the start of the experimental treatments (Supplementary Figure S1A). On March 20, 2011 and through the remainder of the experimental period, we observed a coral bleaching event on the reef (**Figure 1** photograph). **Figure 2** integrates details of the environmental setting of this study with observations of *in situ* bleaching on the reef flat and our experimental design.

Once acclimated to flow-through tanks, coral branches were subjected to a variety of experimental injection treatments (e.g., viral inoculate, heat-killed viral inoculate, not injected) and some *A. aspera* were additionally exposed to a thermal stress treatment (∼2°C above ambient) and/or virus-free seawater inoculation; these treatments are described in the Supplementary Material. Following this, every 24 h, all coral branches were photographed and monitored for signs of stress and/or disease. The visual appearance of each coral branch, as well as the presence/absence of mucus, bleaching, and lesions were recorded. On March 25, 2011, 40 *A. aspera* specimens were sacrificed for TEM and five control saline-injected *A. aspera* samples were frozen at –80°C for virome analysis. Different fragments were used for microscopic and genomic analysis, but these fragments were from the same parent colonies and experienced identical treatments. On March 27, 2011, 12 *A. millepora* specimens were sacrificed for TEM.

### Transmission Electron Microscopy

Approximately 1 to 5 mm^3^ per coral specimen was immersed in a TEM fixative (2% EM-grade glutaraldehyde in virus-free 3x PBS) and stored in the dark at 4°C until processing. Samples were processed following the methods of Le Tissier (1991). In brief, decalcified coral samples (20% ETDA) were washed in 0.2 M cacodylate buffer (pH 7.0) and post-fixed with 1% osmium tetroxide in 0.1 M cacodylate buffer (pH 7.0). The samples were subsequently washed in distilled water, dehydrated through immersion in a series of ethanol and propylene oxide baths, and embedded in resin. Ultra-thin sections were generated using a Leica ultracut UC6 microtome and diamond knife. Sections were stained on 200 μm copper grids for 3 minutes with 5% aqueous uranyl acetate followed by a 1-min stain with lead citrate. Replicated tissue sections were collected from each resin-blocked sample until all coral tissue regions and layers could be assessed. Multiple sections from each sample were visualized; each sample was viewed for equal time (approximately 1 h) on a JEM-1010 Transmission Electron Microscope at the University of Queensland Centre for Microscopy and Microanalysis.

### Virome Generation and Analysis

A single virome was constructed from viral DNA from five control saline-injected *A. aspera* specimens to corroborate TEM data. All fragments appeared healthy (i.e., did not exhibit evidence of paling or tissue sloughing) when frozen at the end of the experiment (Supplementary Figure S1B). With some minor modifications, we used our standard protocol for isolating viral particles from coral tissues (Thurber et al., 2009). Briefly, each coral specimen was defrosted and tissue removed with an airbrush containing virus-free 1x PBS (pH 7.4). This combined slurry was centrifuged at 3220 × *g* for 20 min at room temperature. Samples were decanted and the supernatant passaged through a 0.8 μm nucleopore impact filter from Whatman. Filtrate was placed on a CsCl density gradient containing four densities (1.7, 1.5, 1.35, 1.2 g ml^–1^) and spun at 22,000 rpm for 2 h at 4°C. Abundant viral particles were identified from the 1.2 g ml^–1^ CsCl density layer (Noble and Fuhrman, 1998) and recovered. This viral isolate was then filtered through a 0.45 μm Sterivex. Particle DNA was extracted with a formamide procedure (Thurber et al., 2009) and then amplified using the phi29 polymerase multiple displacement method (Dean et al., 2001; *Methodological Considerations* provided in Supplementary Materials). The Genomiphi kit V2 (GE Healthcare Life Sciences) was used according the manufacturer’s recommended procedure.

Approximately 16.5 ng of this combined *A. aspera* control saline-injected viral DNA was prepared for sequencing using the Nextera XT kit. Illumina MiSeq 150 bp paired-end sequencing generated reads of approximately 300 bp. Sequences were quality filtered (phred = 30) and trimmed. High quality paired-end reads were merged using PEAR (Zhang et al., 2014). Merged and singleton sequences were then combined into a single file for further analysis. Sequences were screened for host and human contamination by using BLASTn (*e*-value ≤ 10^–20^) against the *Acropora digitifera* and human genomes, respectively. Sequences were further filtered with BLASTn (*e*-value ≤ 10^–20^) to the entire Refseq NCBI database to remove any reads that annotated as potential cellular organisms (i.e., sequence contaminants). Reads with BLASTn annotations to viruses were then assembled with Velvet using a kmer size of 71 (Zerbino and Birney, 2008). A tBLASTx analysis of the contigs was then performed against the NCBI RefSeq viral database (*e*-value ≤ 10^–7^). Viral taxonomy was assigned using NCBI’s taxonomy tree and in-house python scripts. Similarities were then parsed at the viral Family level. Reads were archived at the European Nucleotide Archive (ENA; Accession #PRJEB12107).

## Results

At the end of the experimental period, some coral fragments (particularly those exposed to heat treatments) exhibited visible signs of stress including paling, bleaching, and tissue sloughing. These signs generally coincided with the onset of an *in situ* bleaching event on Heron Island. No potential effects from the control saline injection were evident in any *A. aspera* samples during the experimental period based on daily photographs of each coral fragment and visual inspection of fragments at regular intervals. Regardless of our experimental challenges, all *A. aspera* and *A. millepora* fragments (including non-injected controls) showed microscopic evidence consistent with a massive viral infection. Given this, we chose to generate a single virome from the control saline-injected *A. aspera* samples (*N* = 5 fragments) and we here interpret all TEM data jointly (independent of challenge). Based on these congruent morphological and genomic data, we show that this outbreak consisted of four major viruses: an atypical herpes-like virus, a retrovirus similar to gamma-retroviruses, and two NCLDVs: one 150–180 nm VLP most similar to phycodnaviruses and associated with the host coral and another ∼300–500 nm NCLDV in the candidate Family *Megaviridae* and associated with resident *Symbiodinium*.

### Virome Analysis Reveals Dominance by Diverse Eukaryotic Viruses and Phages

Using viral particle purification and the Illumina MiSeq platform, a 5,069,340 sequence virome library was generated that had a mean sequence length of 301 bp, of which 829,330 (16%) passed quality control and pre-screening for similarities to non-viral targets. Velvet assembly resulted in a total of 70,807 contigs, with a mean length of 1,160 bp and a maximum of 3,070 bp. Of these, 18,432 contigs (26%) were highly similar (i.e., *e*-values ≤ 10^–7^) to a completed viral genome. These contigs contained ∼42,000 viral gene annotations, of which 2,587 were unique. Of these, 841 unique contigs fell within viral Families.

**FIGURE 3:**
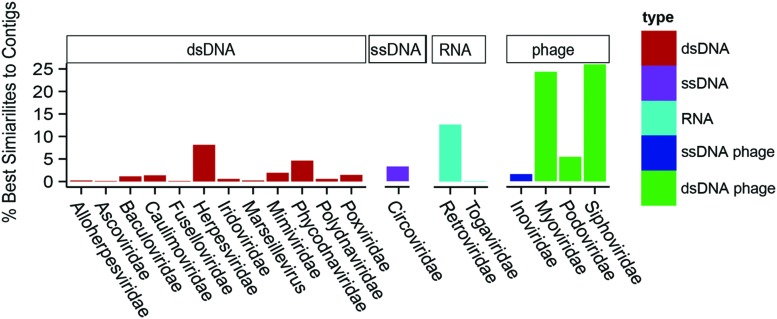
**Relative percentage of viral Families found in a single control *Acropora aspera* coral virome (generated from five coral fragments) using the best tBLASTx similarities to assembled contigs**. Bar heights indicate the relative percentages of best similarities to viral Families (eukaryotic viruses) or categories of phages. Colors of bars distinguish phages (dark blue and green) from eukaryotic viruses, and genome type within the eukaryotic viruses (red = dsDNA, purple = ssDNA, light blue = RNA).

To determine the types of viruses present in *Acropora aspera*, unique contig similarities were binned hierarchically based on annotations into 19 viral Families (**Figure 3**). Overall, these contigs were similar to phages and three groups of eukaryotic viruses (dsDNA, ssDNA, and RNA genomes). A majority of the annotations fell within the Group I classification of dsDNA eukaryotic viruses (**Figure 3**, red bars), retroviruses (light blue bars) and phages (green and dark blue bars). The five dominant families were: *Siphoviridae*, *Myoviridae*, *Retroviridae*, *Herpesviridae*, and *Phycodnaviridae*, in decreasing order of relative abundance. Family-level analyses based on the (1) top five similarities to a given contig, (2) top read hits, and (3) top five similarities to a given read produced results concordant with the top contig results described here (Supplementary Tables S1A,B). Three ssDNA viral Families, the *Circoviridae*, *Inoviridae*, and *Microviridae*, produced many more similarities to reads than to contigs (see *Methodological Considerations* in Supplementary Material, Supplementary Table S1A).

### Herpesvirus-Like Viral Particles are Abundant in *Acropora aspera* and *Acropora millepora* Corals

**FIGURE 4:**
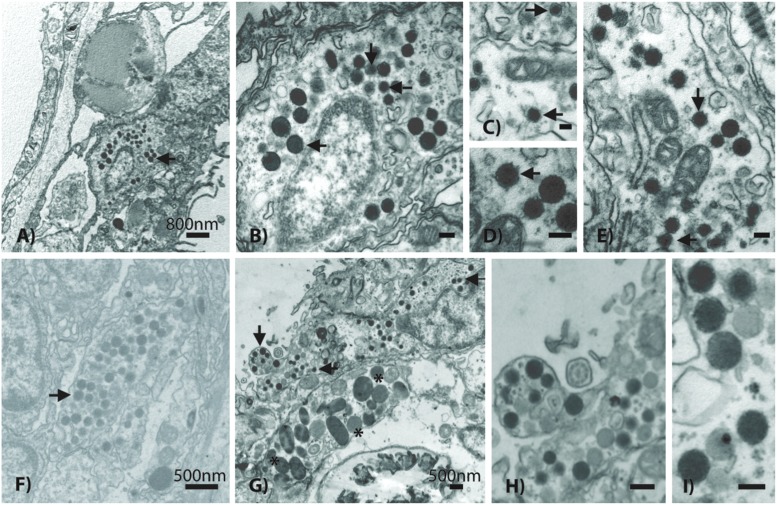
**Representative examples of the acroporid atypical herpes-like virus from *Acropora aspera* coral fragments**. These abundant herpes-like VLPs are comprised of enveloped, icosahedral (non-tailed) capsids ranging from 120 to 150 nm in diameter and contain electron dense cores. The VLPs in **(F)** are within a cellular vacuole; this is characteristic of herpesviruses. **(B)** is an enlargement of **(A)**; **(D)** is an enlargement of **(E)**; **(H)** is an enlargement of **(G)**. **(A,B)** and **(G–I)** are images of the control saline-injected coral treatment fragments used to generate the viral metagenome. Arrows in **(A–G)** indicate general examples of atypical herpes-like viral particles. Asterisks indicate bacterial cells. Scale bars are 100 nm, unless otherwise noted.

Based on TEM evidence, the most commonly identified VLP in both coral species was composed of an enveloped and circular capsid ranging from 120 to 150 nm in diameter and containing an electron dense core (**Figure 4**), a morphology highly reminiscent of herpesviruses. These herpes-like VLPs were present individually and as large clusters (**Figures 4A,B,F**) that ranged from ∼5 to 40 VLPs within host coral epidermal and gastrodermal cells. *Acropora aspera* contained most of the large inclusions of this VLP. In some instances, clusters of these VLPs were found in cellular vacuoles (**Figures 4F–H**) similar to those commonly found in herpes infections (Schmid et al., 2014). In vacuoles, herpes-like VLPs often were associated with other VLPs (**Figures 4G,H**) and constituted the dominant structure within host cells (**Figure 4F**). Herpes-like VLPs were not observed in host cell nuclei.

Results from the *A. aspera* virome were consistent with the observation of herpes-like VLPs in TEM images. The third most abundant unique eukaryotic virus similarities (72 unique contig and 816 read annotations) were to the *Herpesvirales* Order (*Herpesviridae* and *Alloherpesviridae*; **Figure 3**). Similarities to several important functional genes were characterized, including: a uracil DNA glycosylase and a DNA polymerase (**Table 1**). Further, when considering all contig annotations (not just unique ones), a total of 15,083 contigs were similar to a single *betaherpesvirinae* genome, Human herpesvirus 6A. Phylogenetic analysis of a DNA polymerase-like contig generated in this study indicates that it originates from an undescribed virus within the *Herpesviridae* that is most similar to mammalian gammaherpesviruses (methods and detailed results provided in the Supplementary Material, Supplementary Figure S2).

**Table 1:** Examples of gene annotations from a single control *Acropora aspera* virome (generated from five coral fragments), including those from *Herpesviridae*, the Nucleocytoplasmic Large DNA Viruses (NCLDVs), and *Retroviridae*.

Viral family annotation	ENA Accession No.	Contig coverage	nt Size	*E*-Value	aa % Identity	Viral genome similarity	Gene annotation
*Herpesviridae*	LT009377	6.43	511	2*e* – 44	52%	Elephant endotheliotropic herpesvirus 6	Uracil DNA glycosylase
*Herpesviridae*	LT009378	1.18	497	2*e* – 17	34%	Gorilla lymphocryptovirus 2	DNA polymerase
NCLDV/Mega	LT009379	2.5	635	2*e* – 11	28%	*Pyramimonas orientalis* virus	MutS protein
NCLDV/Mega	LT009380	1.34	321	4*e* – 53	64%	Lausannevirus	Eukaryotic peptide chain release factor subunit 1
NCLDV/Mega	LT009381	2	907	2*e* – 08	30%	*Acanthamoeba polyphaga* mimivirus	Putative serine/threonine-protein kinase/receptor
NCLDV/Mega	LT009382	5.1	896	1*e* – 34	36%	*Megavirus chilensis*	UDP-*N*-acetylglucosamine 2-epimerase
NCLDV/Mega	LT009383	1.94	736	2*e* – 10	49%	*Cafeteria roenbergensis* virus BV-PW1	Superfamily II helicase/eIF-4AIII
NCLDV/Mega	LT009384	2.45	751	2*e* – 22	45%	*Cafeteria roenbergensis* virus BV-PW1	Putative photolyase
NCLDV/Mega	LT009385	1.58	259	7*e* – 21	67%	*Cafeteria roenbergensis* virus BV-PW1	Putative DnaK/Hsp70
NCLDV/Phyco	LT009386	2.37	1209	3*e* – 13	31%	*Bathycoccus* sp. RCC1105 virus	UDP-*N*-acetylglucosamine *O*-acyltransferase
NCLDV/Phyco	LT009387	5.75	720	7*e* – 09	47%	*Paramecium bursaria Chlorella* virus	ATP-dependent helicase
NCLDV/Pox	LT009388	1.76	880	3*e* – 09	31%	Pigeonpox virus	Hypothetical protein fep_013
*Retroviridae*	LT009389	1.1	666	3*e* – 07	26%	Walleye epidermal hyperplasia virus 1	RT_ZFREV_like reverse transcriptases
*Retroviridae*	LT009390	2.38	474	6*e* – 19	40%	Walleye epidermal hyperplasia virus 2	Polymerase protein
*Retroviridae*	LT009391	4.7	856	5*e* – 15	32%	Reticuloendotheliosis virus	*gag* protein
*Retroviridae*	LT009392	3.2	474	6*e* – 13	46%	Reticuloendotheliosis virus	Envelope glycoprotein
*Retroviridae*	LT009393	4.86	461	2*e* – 14	48%	Reticuloendotheliosis virus	Protease
*Retroviridae*	LT009394	2.18	466	2*e* – 11	28%	Simian foamy virus	Integrase core domain

*Annotations are based on tBLASTx to the NCBI viral database (e-value threshold of <10^–7^). Contig sequences can be obtained through the European Nucleotide Archive (ENA).*

### NCLDV-Like Viruses in Host Tissues

**FIGURE 5:**
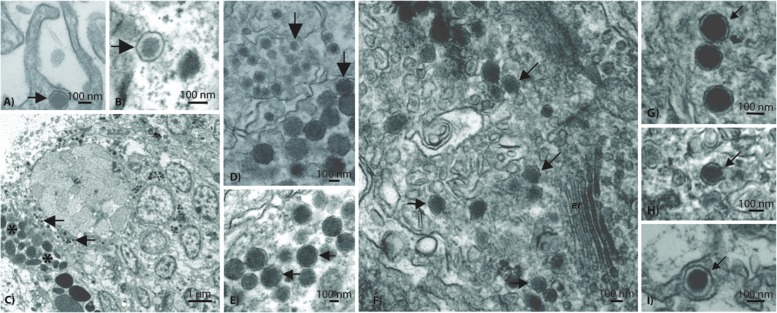
**Representative examples of a phycodnavirus-like nucleocytoplasmic large DNA virus (NCLDV) in *Acropora aspera* (**A–E)** and *Acropora millepora***(F–I)** coral fragments**. Asterisks indicate bacterial cells. Scale bars are as follows: **(A,B,D–I)** = 100 nm; **(C)** = 1 μm.

The next most common VLP type observed in both *A. aspera* and *A. millepora* epidermal cells fell within the NCLDVs (**Figure 5**). In TEM images where this type was observed, 10 to 59 VLPs were typically present. This VLP morphology was icosahedral, electron-dense and enveloped. However, these VLPs were consistently larger in diameter (150–180 nm) than the 120–150 nm herpes-like virus. Very dark membranes, a more angular appearance, and a wider space between the electron-dense core and the envelope membrane (**Figures 5B,I**) also distinguished these VLPs, relative to the herpes-like viruses.

Within the *A. aspera* virome, the second most abundant group of eukaryotic virus annotations was to the NCLDVs (79 unique contigs and 733 read annotations). These unique similarities fell among the Families that make up the candidate NCLDV cluster: *Phycodnaviridae* (*n* = 41 best contigs), *Mimiviridae* (*n* = 17), *Poxviridae* (*n* = 13), *Iridoviridae* (*n* = 5), *Marseillevirus* (*n* = 2), and *Ascoviridae* (*n* = 1). Importantly, these annotations contain multiple phylogenetically informative protein encoding genes including: an ATP-dependent helicase, a UDP-*N*-acetylglucosamine *O*-acyltransferase, a peptide release factor, a DnaK/Hsp70, and a putative photolyase (**Table 1**). When analyzed based on all contig annotations (not just unique ones) the most abundant similarities to this classification were to four genomes within the candidate Family *Megaviridae* and one *Phycodnaviridae* genome: *Acanthamoeba polyphaga* mimivirus (*n* = 50), *Megavirus chilensis* (*n* = 28), *Cafeteria roenbergensis* virus (*n* = 22), *Phaeocystis globosa* virus (*n* = 14), and *Paramecium bursaria Chlorella* virus (*n* = 50), respectively.

### *Symbiodinium*-Associated Megaviruses

**FIGURE 6:**
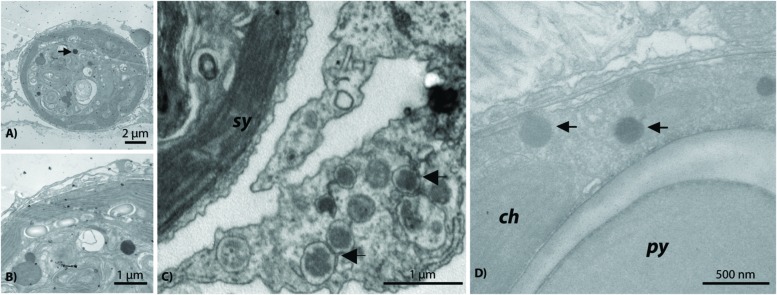
**Representative examples of the giant *Megaviridae*-like NCLDV observed within *Symbiodinium* cells residing in *Acropora aspera***(A,B)** and *Acropora millepora***(D)** and near *Symbiodinium* cells in a probable *A. aspera* symbiosome **(C)****. *ch*, chloroplast; *py*, pyrenoid; *sy*, *Symbiodinium*.

In addition to the ∼150 nm NCLDV described above, another NCLDV-like icosahedral VLP was observed within and adjacent to probable symbiosomes (e.g., **Figure 6C**). These *Symbiodinium*-associated VLPs had mean diameters from 300 to 500 nm but varied in capsid morphology with some being rounded (**Figures 6A,B**) and others polyhedral (**Figure 6C**). VLPs in this size range have been referred to as megaviruses, and several of the NCLDV viral Families (e.g., *Mimiviridae*, *Marseillevirus*) fall within this size category (Monier et al., 2008; Claverie et al., 2009). Given their proximity to or location within *Symbiodinium* cells, these putative viruses are a potentially distinct nucleocytoplasmic large DNA viral type perhaps within *Phycodnaviridae* or candidate Family *Megaviridae*. For example, although numerous gene annotations were found across these Families, one phylogenetically informative sequence, MutS, indicated that this putative virus is most similar to *Pyramimonas orientalis* virus (**Table 1**), a genome recently reclassified as a megavirus (Legendre et al., 2014; Moniruzzaman et al., 2014). Similarly, phylogenetic analysis of this contig indicated that it originated from an undescribed virus within the *Megaviridae* (for methods and detailed results, see Supplementary Material; Supplementary Figure S3).

### Morphological and Metagenomic Evidence of Additional Acroporid Virus Diversity

**FIGURE 7:**
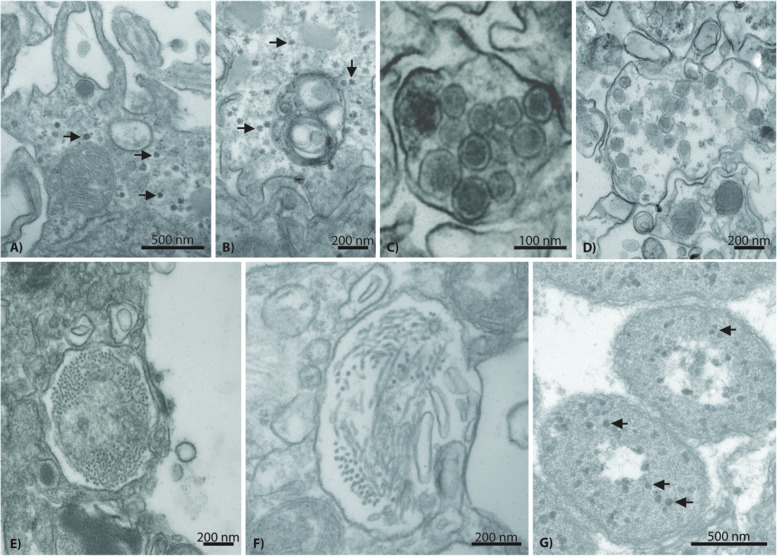
**Representative examples of additional VLP diversity observed in *Acropora aspera* and *Acropora millepora***. In **(A,B)**, arrows indicate star-shaped VLPs that are morphologically reminiscent of circovirus and nanovirus particles. **(C,D)** Depict retrovirus-like VLPs; the VLPs in **(C)** are within a cell vacuole. **(E,F)** Appear to be long filamentous VLPs within large ovoid viral factories. In **(G)**, arrows indicate phage-like VLPs within bacterial cells (large, ovoid polygons) enclosed in peri-algal spaces of the host gastroderm. Scale bars are 200 nm, unless otherwise noted.

Additional VLP morphologies were identified in all corals, but appeared to be present at lower prevalence than the herpes-like and NCLDV-like VLP types described above. These other putative viral types included small (∼17–40 nm) star/pentagonal shaped viruses similar to astro-, circo-, parvo-, or nano-like virus particles (**Figures 7A,B**), amorphous or egg-shaped retrovirus-like VLPs (∼70 nm in diameter; **Figures 7C,D**), filamentous VLPs within large viral factories or inclusion bodies (**Figures 7E,F**), and phage-like VLPs within bacterial aggregates enclosed in peri-algal spaces of the host gastroderm (**Figure 7G**).

In the *A. aspera* virome, phage sequences were dominated by the *Sipho-* and *Myoviridae* (**Figure 3**) with the top two most abundant contig annotations being similar to a *Streptococcus* myophage (*n* = 616 total) and a *Streptococcus* siphophage (*n* = 298). These large numbers of sequences are notable because the next most abundant group of phage annotations was to a *Pelagibacter* phage, which only had 28 total similarities across all contigs. Sequences with similarity to the *Retroviridae* were highly abundant in the *A. aspera* virome; *Circoviridae* were also notable (<5%; **Figure 3**).

## Discussion

Using a modification and combination of established methods for coral viral metagenomics and TEM, we found highly congruent molecular (1000s of high quality sequences and contigs) and physical (1000s of VLPs) evidence to support the hypothesis that although a diverse group of viruses are present in acroporid corals, four main or core eukaryotic viruses dominate these species: two NCLDVs including a new megavirus, a somewhat atypical herpes-like virus, and a gamma-retrovirus. It should be noted that genomic support for these four core viral groups are based on metagenomic analyses of control saline-injected fragments of *A. aspera* that showed a high abundance of diverse VLPs in the TEM images. Although sequencing of additional samples may have produced some novel evidence of viral diversity in terms of similarities to diagnostic viral genes or the construction of longer contigs, it is unlikely that it would have profoundly influenced the core viral groups identified from these two acroporid species. We infer this based on the fact that TEM images generated from all experimental corals were examined, and all major VLP morphologies from the total dataset were linked to predominant viral sequence similarities in the metagenome. Thus, to a certain extent, joint application of microscopy and metagenomics (even to a single treatment) improved our confidence in the identification of the dominant or core viral types within an environmental consortium.

### Multiple Nucleocytoplasmic Large DNA Viruses Infect Coral Holobionts

Nucleocytoplasmic large DNA virus sequences have been found in every coral virus metagenomic study undertaken thus far (Wood-Charlson et al., 2015) and often are the most abundant similarities found in corals and in cultures of their dinoflagellate algal symbionts (Wegley et al., 2007; Correa et al., 2013; Weynberg et al., 2014). Two distinct variants reminiscent of NCLDVs were commonly identified in this study. One variant was a ∼150 nm NCLDV located within host tissues (**Figure 5**); the other variant was significantly larger at ∼300 nm and located near or within *Symbiodinium* (**Figure 6**). Congruently, NCLDV contig similarities were highly abundant in the virome we generated. Work on NCLDVs is rapidly advancing, and new viruses are now found routinely (e.g., pithovirus, Legendre et al., 2014; faustovirus, Reteno et al., 2015). NCLDVs span a large range of particle sizes (∼140–1,100 nm diameter capsids) and genome lengths (∼100–2.5 Mb; King et al., 2012; Colson et al., 2013). Thus ascribing gene sequences within the systematic framework of this group of viruses remains problematic due to the vagaries of viral morphologies and genome sequences. However, the microscopic and Family-level genomic data generated here (**Figure 3**), in combination with a review of the literature and an in-depth phylogenetic analysis of sequences from this virome and other previously annotated viromes (e.g., Correa et al., 2013), provide us with sufficient data to clearly delineate the taxonomic identity of the ∼150 nm versus the ∼300 nm coral-associated NCLDVs.

The identity of the larger (∼300 nm) NCLDV characterized here is almost certainly a new relative of the megaviruses. The most common annotations in the dataset were to members of this candidate Family including: *Acanthamoeba polyphaga* mimivirus, *Megavirus chilensis*, *Cafeteria roenbergensis* virus, and *Phaeocystis globosa* virus strain 16T virus genomes. Importantly, one of the genes identified in our virome was a MutS homolog (**Table 1**); this gene has become diagnostic for megaviruses and has led to a reorganization of this clade of the NCLDVs (Colson et al., 2013; Wilson et al., 2014). In our unrooted tree, this *A. aspera* MutS contig is placed as sister taxa to a clade containing two megaviruses and a phycodnavirus with strong bootstrap support (Supplementary Figure S3). Further, re-annotation of our previously identified viral mRNA sequences (GenBank # JX026066.1; Correa et al., 2013) from both a coral and a *Symbiodinium* culture showed that another diagnostic gene for this candidate Family, DNA Polymerase B, also annotates as *Pyramimonas orientalis* virus (BLASTx 66% identity, *e*-value ≤ 8*e*^–58^). Given that gene similarities to these viruses have now been found in corals from the Atlantic and Pacific, as well as in *Symbiodinium* cultures, they likely represent a cosmopolitan *Symbiodinium*-infecting megavirus.

Given the physical size of these VLPs imaged in and adjacent to *Symbiodinium* (**Figure 6**) and the identified MutS gene similarity in our *A. aspera* virome, we hypothesize that coral holobionts harbor megaviruses most likely related to the *Pyramimonas orientalis* virus (Sandaa et al., 2001) or perhaps *Cafeteria roenbergensis* virus (Fischer et al., 2010). However, megaviruses vary in their physical structure both in size and shape (e.g., the presence/absence of projections; Fischer et al., 2010). Interestingly, *Cafeteria roenbergensis* virus, like many mimiviruses, contains a unique star-like structure at one end of the virion (Colson et al., 2011), whereas *Pyramimonas orientalis* virus does not (Sandaa et al., 2001). As mentioned above, after re-analysis of viral mRNA sequences from our previous work on NCLDVs in corals and *Symbiodinium*, we found that another diagnostic gene for this candidate Family (Takemura et al., 2015), DNA polymerase B, also annotates to *Pyramimonas orientalis* virus. Therefore, given that: (1) our NCLDV lacks a star-shaped structure and is genetically similar to the *Pyramimonas orientalis* clade of megaviruses based on phylogenetic reconstruction of a diagnostic gene (MutS), and (2) similarities to this viral clade have previously been recovered from both corals and *Symbiodinium* cultures (Correa et al., 2013), the most parsimonious interpretation of these physical and genetic data is that the *Symbiodinium*-associated megavirus in this study is a cosmopolitan relative of the *Pyramimonas orientalis* megaviruses.

In contrast, the identity of the 150 nm NCLDV particles described here is less straightforward. Importantly, these putative viruses are not reminiscent of the originally described mimiviruses (La Scola et al., 2003; C. Desnues, personal communication), but more similar to phycodnaviruses and iridoviruses (King et al., 2012), as well as megaviruses (Sandaa et al., 2001) in that they lack the characteristic capsid hair-like projections of mimiviruses (**Figure 5**). Many phylogenetically relevant genes within the *Phycodnaviridae* were annotated in this work (**Table 1**). For example, 50 unique contig similarities were found to a single member, *Paramecium bursaria Chlorella* virus NY2A, of this phycovirus Family. Although phycodnaviruses are not generally thought to associate with multicellular eukaryotes, recent evidence has shown that they can infect non-algal hosts, including humans (Yolken et al., 2014). Thus, we suggest that these *Phycodnaviridae* similarities represent a phycovirus relative that infects the coral host. An alternative, but unlikely explanation, is that these 150 nm viruses do infect *Symbiodinium* as they are similar to those in Lawrence et al. (2014) but were only visualized moving through the host *en route* to the free-living environment.

### Atypical Herpes-Like Viruses in Corals

A major interest and perplexing aspect of this work, was the high prevalence of the ∼120 nm VLPs that were morphologically similar to and biologically different from herpesviruses. In this study, VLPs of this average size were visually indistinguishable from many herpesviruses in terms of their capsid size and envelope. However, described herpesviruses predominantly replicate in the nuclei of cells (Schmid et al., 2014), whereas herpes-like particles in this study were never identified in the nuclei of coral cells. Thus, we hypothesize that the ∼120 nm VLPs described here are not true herpesviruses, but perhaps something distinct.

Further, although we (Vega Thurber et al., 2008; Vega Thurber and Correa, 2011; Soffer et al., 2014a) and others (Marhaver et al., 2008; Weynberg et al., 2014) have found metagenomic evidence that herpes-like annotations can dominate dsDNA viral taxa in corals, these similarities align with only a few coding regions of herpesvirus genomes, have low percent identity to known herpesviruses, and rarely span large portions of the herpesvirus genomes (see Wood-Charlson et al., 2015). In the unrooted DNA polymerase gene tree, the *A. aspera* contig is placed in a clade containing all primate taxa in the phylogeny except the squirrel monkey, *Saimiri sciureus* (Supplementary Figure S2). The exact position of the *A. aspera* DNA polymerase contig within this clade could not be resolved, but the sequences generated in this study appear to be relatively distinct from previously sequenced herpesvirus DNA polymerase genes. Thus we hypothesize that these viruses are ‘atypical’ in that they are highly morphologically reminiscent of herpesviruses, but only marginally similar to herpesviruses at the genomic and cell cycle levels. What these novel viruses truly are and how they affect their host corals remains an intriguing question. Future investigations should aim to evaluate the genomes of these viruses, perhaps by using deeper and longer sequencing approaches (e.g., PacBio), cell culture-based work, or size selection-based flow sorting (Martinez et al., 2014).

### Multiple Lines of Evidence for Retroviruses in Acroporid Corals

Retrovirus-like sequence similarities have previously been characterized from stony corals. For example, they comprised 6.8 and 10%, respectively, of sequence similarities obtained from control and heat-stressed viromes generated from the Caribbean coral, *Montastraea cavernosa* (Correa et al., 2013). The number of retrovirus-like sequence similarities recovered in this study from *Acropora aspera* (12.7%) is thus comparable to previous findings. Although VLPs physically similar to retroviruses were observed in the *Acropora aspera* samples used for virome generation, they were encountered relatively infrequently (**Figures 7C,D**). These particles were ∼70 nm in diameter with a somewhat amorphous or egg-shaped morphology reminiscent of amphotropic retroviruses (Schlaberg et al., 2009; **Figure 1** on page 477 of King et al., 2012; Pollock et al., 2014). Yet, the most abundant set of unique eukaryotic virus similarities were to the *Retroviridae* (*n* = 112 best contig annotations, **Figure 3**). These annotations included all the structural genes specifically important to this group including *gag*, *pro*, *pol*, and *env*, as well as non-structural genes, such as an integrase (**Table 1**). A majority (73%) of these annotations were from gammaretroviruses most similar to the Reticuloendotheliosis virus and Porcine type-C oncovirus genomes. The next most abundant (12.6%) of these annotations were to a spumavirus, Simian foamy virus. Several of these annotations were to a special group of reverse transcriptases in these viruses that contain RT_ZFREV_like domains (**Table 1**) that are only found in true retroviruses and not retro-elements, confirming their viral origin (Shen and Steiner, 2004).

With regards to RNA viral diversity, it should be noted that we only enriched for DNA viruses; any RNA virus that does not have an intermediate DNA stage would have been missed. If the small (∼17–40 nm) and abundant filamentous viruses cataloged in a few of the TEMs (**Figures 7E,F**) were RNA-based as hypothesized previously (Lohr et al., 2007; Correa et al., 2013; Weynberg et al., 2014; Wood-Charlson et al., 2015), then the virome data would tell us little about their genomic identity. In a previous effort to enrich for RNA genomes (Correa et al., 2013), we identified five sequence similarities to *Heterocapsa circularisquama* RNA virus (HcRNAV), a +ssRNA virus that infects free-living dinoflagellates. However, sequence similarities to HcRNAV were not observed in this study and, overall, there remains little molecular data to suggest that RNA viruses similar to previously described groups are a major component of the acroporid coral virome. Additional work should be conducted in this capacity.

### Viral Outbreak in Corals Associated with a Reef Flat Bleaching Event

This study characterizes the viral consortia from acroporid colonies that: (1) experienced aerial exposure and hyposmotic stress *in situ* on the reef flat, (2) were collected and acclimated to flow-through experimental tanks, and then (3) experienced experimental injection and heat (*A. aspera* only) treatments. Microscopic and genomic data were then interpreted from these samples. No experimental corals were bleached at the time of collection or at the start of the experimental treatments, yet the collected coral colonies did experience abiotic stressors on the reef flat prior to collection for this experiment (**Figure 2**, Supplementary Material). These stressors could have primed or triggered viral lytic cycles in the experimental corals that were only evident at the end of the experimental period (in TEM and genomic investigations). VLP morphologies representative of all predominant groups identified from the metagenome were observed in all experimental treatments (including non-injected controls), and no potential effects from injection treatments were visually evident in any coral fragments. Thus, we infer that the documented viral outbreak most parsimoniously reflects a common response among all experimental corals to aerial exposure and hyposaline (rainfall) stressors on the reef prior to collection for this study, and is not likely driven by our experimental treatments.

We interpret this event as an outbreak for several reasons. First, we identified a diversity of viruses from a relatively small number of samples and sequencing effort, yet observed VLP abundances that were two to three times higher than those previously reported from *Acropora muricata* at Heron Island and Lizard Island, Great Barrier Reef (i.e., 2 to 20 VLPs per cell or membrane-bound vacuole for all VLP size ranges; Patten et al., 2008). Further, similar viruses were detected from all experimental fragments of two different acroporid species (based on TEM). Relative to experimental corals, conspecific acroporids that remained on the reef flat during the study period experienced additional episodes of aerial exposure and coincident intense rainfall (on March 21st–22nd), which likely triggered the observed massive bleaching (e.g., Baker and Cunning, 2015). Thus, we hypothesize that our experimental corals and many Heron Island reef flat acroporids had high viral loads simultaneously.

## Conclusion

The combined application of physical and genomic-based methods in this study provided some significant benefits over using either approach in isolation. Since the TEMs and virome generated in this experiment contained evidence of numerous and diverse putative viruses, we suspect that environmental conditions (low tide-driven aerial exposure coincident with hyposaline conditions due to heavy rainfall) on the reef flat led to mass bleaching on the Heron Island reef flat in March of 2011, which was associated with a viral outbreak. This suggests that stressful environmental conditions can rapidly trigger the onset of viral infection by multiple etiological agents (e.g., atypical herpes-like virus, NCLDV-like viruses including megaviruses) concurrently, and highlights our uncertainty regarding the disease signs exhibited in coral viral infections. Future studies should explore whether increased viral loads are ubiquitous in bleached corals regardless of the stressor triggering bleaching.

## Author Contributions

AC, AT, and RV designed and implemented the study, and collected data in the field. TA performed the microscopy, and AC, TA and RV interpreted the microscopy results. CB created the viral metagenome; SR and RV analyzed it; and AC, SR, and RV interpreted the metagenome results. AC, TA, RS, and RV wrote the manuscript. All authors edited the manuscript.

## Conflict of Interest Statement

The authors declare that the research was conducted in the absence of any commercial or financial relationships that could be construed as a potential conflict of interest.

## Footnotes

1 “NOAA Declares Third Ever Global Coral Bleaching Event.” *National Oceanic and Atmospheric Administration*. NOAA, October 8, 2015. Web. January 12, 2016.

2 http://www.imos.org.au/

## References

[B1] Baker A. C. Cunning R. (2015). “Coral “bleaching” as a generalized stress response to environmental disturbance,” in *Diseases of Coral*, eds Woodley C. M. Downs C. A. Bruckner A. W. Porter J. W. Galloway S. B. (Hoboken, NJ: John Wiley & Sons, Inc.), 396–409.

[B2] Bettarel Y. Bouvier T. Nguyen H. K. Thu P. T. (2015). The versatile nature of coral-associated viruses. *Environ. Microbiol.* 17 3433–3439. 10.1111/1462-2920.12579 25171444

[B3] Bettarel Y. Thuy N. T. Huy T. Q. Hoang P. K. Bouvier T. (2013). Observation of virus-like particles in thin sections of the bleaching scleractinian coral *Acropora cytherea*. *J. Mar. Biol. Assoc.* 93 909–912. 10.1017/S0025315411002062

[B4] Claverie J. M. Grzela R. Lartigue A. Bernadac A. Nitsche S. Vacelet J. (2009). *Mimivirus* and Mimiviridae: giant viruses with an increasing number of potential hosts, including corals and sponges. *J. Invertr. Pathol.* 101 172–180. 10.1016/j.jip.2009.03.011 19457438

[B5] Colson P. De Lamballerie X. Yutin N. Asgari S. Bigot Y. Bideshi D. K. (2013). “Megavirales,” a proposed new order for eukaryotic nucleocytoplasmic large DNA viruses. *Arch. Virol.* 158 2517–2521. 10.1007/s00705-013-1768-6 23812617 PMC4066373

[B6] Colson P. Gimenez G. Boyer M. Fournous G. Raoult D. (2011). The giant Cafeteria roenbergensis virus that infects a widespread marine phagocytic protist is a new member of the fourth domain of life. *PLoS ONE* 6:e18935 10.1371/journal.pone.0018935 PMC3084725 21559486

[B7] Correa A. M. S. Welsh R. M. Thurber R. L. V. (2013). Unique nucleocytoplasmic dsDNA and +ssRNA viruses are associated with the dinoflagellate endosymbionts of corals. *ISME J.* 7 13–27. 10.1038/ismej.2012.75 22791238 PMC3526182

[B8] Davy J. E. Patten N. L. (2007). Morphological diversity of virus-like particles within the surface microlayer of scleractinian corals. *Aquat. Microb. Ecol.* 47 37–44. 10.3354/ame047037

[B9] Dean F. B. Nelson J. R. Giesler T. L. Lasken R. S. (2001). Rapid amplification of plasmid and phage DNA using phi29 DNA polymerase and multiply-primed rolling circle amplification. *Genome Res.* 11 1095–1099. 10.1101/gr.180501 11381035 PMC311129

[B10] De’Ath G. Fabricius K. E. Sweatman H. Puotinen M. (2012). The 27-year decline of coral cover on the great barrier reef and its causes. *Proc. Natl. Acad. Sci. U.S.A.* 109 17995–17999. 10.1073/pnas.1208909109 23027961 PMC3497744

[B11] Fischer M. G. Allen M. J. Wilson W. H. Suttle C. A. (2010). Giant virus with a remarkable complement of genes infects marine zooplankton. *Proc. Natl. Acad. Sci. U.S.A.* 107 19508–19513. 10.1073/pnas.1007615107 20974979 PMC2984142

[B12] Gardner T. Cote I. Gill J. Grant A. Watkinson A. (2003). Long-term region-wide declines in Caribbean corals. *Science* 301 958–960. 10.1126/science.1086050 12869698

[B13] Iyer L. A. Balaji S. Koonin E. V. Aravind L. (2006). Evolutionary genomics of nucleo-cytoplasmic large DNA viruses. *Virus Res.* 117 156–184. 10.1016/j.virusres.2006.01.009 16494962

[B14] King A. M. Q. Adams M. J. Carstens E. B. Lefkowitz E. J. (2012). *Virus Taxonomy: Classification and Nomenclature of Viruses: Ninth Report of the International Committee on Taxonomy of Viruses*. San Diego, CA: Elsevier Academic Press.

[B15] La Scola B. Audic S. Robert C. Jungang L. de Lamballerie X. Drancourt M. (2003). A giant virus in amoebae. *Science* 299:2033 10.1126/science.1081867 12663918

[B16] Lawrence S. A. Wilkinson S. P. Davy J. E. Arlidge W. N. S. Williams G. J. Wilson W. H. (2015). Influence of local environmental variables on the viral consortia associated with the coral *Montipora capitata* from Kaneohe Bay, Hawaii, USA. *Aquat. Microb. Ecol.* 74 251–262. 10.3354/ame01743

[B17] Lawrence S. A. Wilson W. H. Davy J. E. Davy S. K. (2014). Latent virus-like infections are present in a diverse range of *Symbiodinium* spp. (Dinophyta). *J. Phycol.* 50 977–997. 10.1111/jpy.12242 26988781

[B18] Legendre M. Bartoli J. Shmakova L. Jeudy S. Labadie K. Adrait A. (2014). Thirty-thousand-year-old distant relative of giant icosahedral DNA viruses with a pandoravirus morphology. *Proc. Natl. Acad. Sci. U.S.A.* 111 4274–4279. 10.1073/pnas.1320670111 24591590 PMC3964051

[B19] Leruste A. Bouvier T. Bettarel Y. (2012). Enumerating viruses in coral mucus. *Appl. Environ. Microbiol.* 78 6377–6379. 10.1128/AEM.01141-12 22729548 PMC3416620

[B20] Le Tissier M. D. A. (1991). The nature of the skeleton and skeletogenic tissues in the Cnidaria. *Hydrobiologia* 21 397–402. 10.1007/BF00026492

[B21] Littman R. A. Willis B. L. Bourne D. G. (2011). Metagenomic analysis of the coral holobiont during a natural bleaching event on the Great Barrier Reef. *Environ. Microbiol. Rep.* 3 651–660. 10.1111/j.1758-2229.2010.00234.x 23761353

[B22] Lohr J. Munn C. B. Wilson W. H. (2007). Characterization of a latent virus-like infection of symbiotic zooxanthellae. *Appl. Environ. Microbiol.* 73 2976–2981. 10.1128/AEM.02449-06 17351090 PMC1892877

[B23] Maier C. De Kluijver A. Agis M. Brussaard C. P. D. Van Duyl F. C. Weinbauer M. G. (2011). Dynamics of nutrients, total organic carbon, prokaryotes and viruses in onboard incubations of cold-water corals. *Biogeosciences* 8 2609–2620. 10.5194/bg-8-2609-2011

[B24] Marhaver K. L. Edwards R. A. Rohwer F. (2008). Viral communities associated with healthy and bleaching corals. *Environ. Microbiol.* 10 2277–2286. 10.1111/j.1462-2920.2008.01652.x 18479440 PMC2702503

[B25] Martinez M. J. Swan B. K. Wilson W. H. (2014). Marine viruses, a genetic reservoir revealed by targeted viromics. *ISME J.* 8 1079–1088. 10.1038/ismej.2013.214 24304671 PMC3996692

[B26] Monier A. Larsen J. B. Sandaa R. A. Bratbak G. Claverie J. M. Ogata H. (2008). Marine *Mimivirus* relatives are probably large algal viruses. *Virol. J.* 5:12 10.1186/1743-422X-5-12 PMC2245910 18215256

[B27] Moniruzzaman M. Lecleir G. R. Brown C. M. Gobler C. J. Bidle K. D. Wilson W. H. (2014). Genome of brown tide virus (AaV), the little giant of the Megaviridae, elucidates NCLDV genome expansion and host-virus coevolution. *Virology* 466 60–70. 10.1016/j.virol.2014.06.031 25035289

[B28] Nguyen-Kim H. Bouvier T. Bouvier C. Hai D. N. Lam N. N. Rochelle-Newall E. (2014). High occurrence of viruses in the mucus layer of scleractinian corals. *Environ. Microbiol. Rep.* 6 675–682. 10.1111/1758-2229.12185 25756121

[B29] Noble R. T. Fuhrman J. A. (1998). Use of SYBR Green I for rapid epifluorescence counts of marine viruses and bacteria. *Aquat. Microb. Ecol.* 14 113–118. 10.3354/ame014113

[B30] Patten N. L. Harrison P. L. Mitchell J. G. (2008). Prevalence of virus-like particles within a staghorn scleractinian coral (*Acropora muricata*) from the Great Barrier Reef. *Coral Reefs* 27 569–580. 10.1007/s00338-008-0356-9

[B31] Pollock F. J. Wood-Charlson E. M. Van Oppen M. J. H. Bourne D. G. Willis B. L. Weynberg K. D. (2014). Abundance and morphology of virus-like particles associated with the coral *Acropora hyacinthus* differ between healthy and white syndrome-infected states. *Mar. Ecol. Prog. Ser.* 510 39–43. 10.3354/meps10927

[B32] Reteno D. G. Benamar S. Khalil J. B. Andreani J. Armstrong N. Klose T. (2015). Faustovirus, an Asfarvirus-related new lineage of giant viruses infecting amoebae. *J. Virol.* 89 6585–6594. 10.1128/JVI.00115-15 25878099 PMC4468488

[B33] Rosario K. Schenck R. O. Harbeitner R. C. Lawler S. N. Breitbart M. (2015). Novel circular single-stranded DNA viruses identified in marine invertebrates reveal high sequence diversity and consistent predicted intrinsic disorder patterns within putative structural proteins. *Front. Microbiol.* 6:696 10.3389/fmicb.2015.00696 PMC4498126 26217327

[B34] Rosenberg E. Zilber-Rosenberg I. (2014). “Microbiotas are part of holobiont fitness,” in *The Hologenome Concept: Human, Animal, and Plant Microbiota*, eds Rosenberg E. Zilber-Rosenberg I. (New York, NY: Springer International Publishing), 55–80.

[B35] Sandaa R. A. Heldal M. Castberg T. Thyrhaug R. Bratbak G. (2001). Isolation and characterization of two viruses with large genome size infecting *Chrysochromulina ericina* (Prymnesiophyceae) and *Pyramimonas* orientalis (Prasinophyceae). *Virology* 290 272–280. 10.1006/viro.2001.1161 11883191

[B36] Schlaberg R. Choe D. J. Brown K. R. Thaker H. M. Singh I. R. (2009). XMRV is present in malignant prostatic epithelium and is associated with prostate cancer, especially high-grade tumors. *Proc. Natl. Acad. Sci. U.S.A.* 106 16351–16356. 10.1073/pnas.0906922106 19805305 PMC2739868

[B37] Schmid M. Speiseder T. Dobner T. Gonzalez R. A. (2014). DNA virus replication compartments. *J. Virol.* 88 1404–1420. 10.1128/JVI.02046-13 24257611 PMC3911613

[B38] Sharma V. Colson P. Giorgi R. Pontarotti P. Raoult D. (2014). DNA-dependent RNA polymerase detects hidden giant viruses in published databanks. *Genome Biol. Evol.* 6 1603–1610. 10.1093/gbe/evu128 24929085 PMC4122926

[B39] Shen C. H. Steiner L. A. (2004). Genome structure and thymic expression of an endogenous retrovirus in zebrafish. *J. Virol.* 78 899–911. 10.1128/JVI.78.2.899-911.2004 14694121 PMC368747

[B40] Soffer N. Brandt M. E. Correa A. M. S. Smith T. B. Vega Thurber R. L. (2014a). Potential role of viruses in white plague coral disease. *ISME J.* 8 271–283. 10.1038/ismej.2013.137 23949663 PMC3906806

[B41] Soffer N. Zaneveld J. Vega Thurber R. L. (2014b). Phage-bacteria network analysis and its implication for the understanding of coral disease. *Environ. Microbiol.* 17 1203–1218. 10.1111/1462-2920.12553 25039472

[B42] Takemura M. Yokobori S. Ogata H. (2015). Evolution of eukaryotic DNA polymerases via interaction between cells and large DNA viruses. *J. Mol. Evol.* 81 24–33. 10.1007/s00239-015-9690-z 26177821

[B43] Thurber R. L. V. Haynes M. Breitbart M. Wegley L. Rohwer F. (2009). Laboratory procedures to generate viral metagenomes. *Nat. Protoc.* 4 470–483. 10.1038/nprot.2009.10 19300441

[B44] van Oppen M. J. H. Leong J. A. Gates R. D. (2009). Coral-virus interactions: a double-edged sword? *Symbiosis* 47 1–8. 10.1007/BF03179964

[B45] Vega Thurber R. L. Barott K. L. Hall D. Liu H. Rodriguez-Mueller B. Desnues C. (2008). Metagenomic analysis indicates that stressors induce production of herpes-like viruses in the coral *Porites compressa*. *Proc. Natl. Acad. Sci. U.S.A.* 105 18413–18418. 10.1073/pnas.0808985105 19017800 PMC2584576

[B46] Vega Thurber R. L. Correa A. M. S. (2011). Viruses of reef-building scleractinian corals. *J. Exp. Mar. Biol. Ecol.* 408 102–113. 10.1016/j.jembe.2011.07.030

[B47] Wegley L. Edwards R. Beltran R.-B. Hong L. Rohwer F. (2007). Metagenomic analysis of the microbial community associated with the coral *Porites astreoides*. *Environ. Microbiol.* 9 2707–2719. 10.1111/j.1462-2920.2007.01383.x 17922755

[B48] Weston A. J. Dunlap W. C. Shick J. M. Klueter A. Iglic K. Vukelic A. (2012). A profile of an endosymbiont-enriched fraction of the coral *Stylophora pistillata* reveals proteins relevant to microbe-host interactions. *Mol. Cell. Proteom.* 11:M111015487. 10.1074/mcp.M111.015487 PMC3433924 22351649

[B49] Weynberg K. D. Voolstra C. R. Neave M. J. Buerger P. Van Oppen M. J. H. (2015). From cholera to corals: viruses as drivers of virulence in a major coral bacterial pathogen. *Sci. Rep.* 5:17889 10.1038/srep17889 PMC4672265 26644037

[B50] Weynberg K. D. Wood-Charlson E. M. Suttle C. A. Van Oppen M. J. H. (2014). Generating viral metagenomes from the coral holobiont. *Front. Microbiol.* 5:206 10.3389/fmicb.2014.00206 PMC4019844 24847321

[B51] Wilson W. H. (2011). “Coral viruses,” in *Studies in Viral Ecology, Volume Two: Animal Host Systems*, ed Hurst C. J. (Hoboken, NJ: John Wiley & Sons, Inc.), 141–149.

[B52] Wilson W. H. Dale A. L. Davy J. E. Davy S. K. (2005). An enemy within? Observations of virus-like particles in reef corals. *Coral Reefs* 24 145–148. 10.1007/s00338-004-0448-0

[B53] Wilson W. H. Francis I. Ryan K. Davy S. K. (2001). Temperature induction of viruses in symbiotic dinoflagellates. *Aquat. Microb. Ecol.* 25 99–102. 10.3354/ame025099

[B54] Wilson W. H. Gilg I. C. Duarte A. Ogata H. (2014). Development of DNA mismatch repair gene, MutS, as a diagnostic marker for detection and phylogenetic analysis of algal *Megaviruses*. *Virology* 466 123–128. 10.1016/j.virol.2014.07.001 25063474

[B55] Wood-Charlson E. M. Weynberg K. D. Suttle C. A. Roux S. Van Oppen M. J. H. (2015). Metagenomic characterization of viral communities in corals: mining biological signal from methodological noise. *Environ. Microbiol.* 17 3440–3449. 10.1111/1462-2920.12803 25708646

[B56] Yamada T. (2011). Giant viruses in the environment: their origins and evolution. *Curr. Opin. Virol.* 1 58–62. 10.1016/j.coviro.2011.05.008 22440568

[B57] Yolken R. H. Jones-Brando L. Dunigan D. D. Kannan G. Dickerson F. Severance E. (2014). Chlorovirus ATCV-1 is part of the human oropharyngeal virome and is associated with changes in cognitive functions in humans and mice. *Proc. Natl. Acad. Sci. U.S.A.* 111 16106–16111. 10.1073/pnas.1418895111 25349393 PMC4234575

[B58] Yutin N. Koonin E. V. (2012). Hidden evolutionary complexity of Nucleo-Cytoplasmic large DNA viruses of eukaryotes. *Virol. J.* 9 1–18. 10.1186/1743-422X-9-161 22891861 PMC3493329

[B59] Zerbino D. R. Birney E. (2008). Velvet: algorithms for de novo short read assembly using de Bruijn graphs. *Genome Res.* 18 821–829. 10.1101/gr.074492.107 18349386 PMC2336801

[B60] Zhang J. Kobert K. Flouri T. Stamatakis A. (2014). PEAR: a fast and accurate Illumina paired-end reAd mergeR. *Bioinformatics* 30 614–620. 10.1093/bioinformatics/btt593 24142950 PMC3933873

