# Supplementary material for: Viral Outbreak in Corals Associated with an In Situ Bleaching Event: Atypical Herpes-Like Viruses and a New Megavirus Infecting Symbiodinium
Source: Front Microbiol. 2016 Feb 22;7:127. doi: 10.3389/fmicb.2016.00127 (PMC4761846; doi:10.3389/fmicb.2016.00127)
Supplement: Supplementary file 1 [file Data_Sheet_1.PDF]

Supplementary Material

Viral outbreak in corals associated with an *in situ* bleaching event: atypical herpes-like viruses and a new megavirus infecting *Symbiodinium*

Adrienne M.S. Correa, Tracy D. Ainsworth, Stephanie M. Rosales, Andrew R. Thurber, Christopher R. Butler, Rebecca L. Vega Thurber*

*** Correspondence:** Rebecca L. Vega Thurber, Oregon State University, Department of Microbiology, 454 Nash Hall, Corvallis, OR, 97331, USA, rvegathurber@gmail.com

1. Supplementary Methods

In 2011, we conducted aquarium-based experiments, henceforth referred to as the 'Aspera' and 'Millepora' experiments, on two acroporid coral species with the intention of inducing viral outbreaks via the injection of viral particles. Unbeknownst to us, the coral reef flat from which we collected coral material for these experiments was in the incipient stages of a bleaching event and (we hypothesize) a viral outbreak, induced by low tide aerial exposure and coincident heavy rainfall. Although all collected corals appeared visually healthy at the start of our controlled experiments, the stressors these colonies experienced *in situ* on the reef obscured any potential treatment-derived effects of our controlled experiments. Thus, in the spirit of full disclosure, we provide the details of our controlled experimental designs and methods below. We do not, however, present the results of this study in terms of these treatments, since their effects were indiscernible in visual observations and photographs of fragments during the experiment, and in subsequent TEM images of these fragments.

Environmental Setting

Preceding the collection of experimental corals and during this study, the Heron Island reef flat experienced a period of low tides that caused repeated aerial exposure of reef flat colonies and increased residence time of water on the reef flat. These events are evident in temperature and tidal height data collected during March of 2011 (Fig. 2). Specifically, the reef flat (measured at the 1.1 m water depth mooring, Fig. 2 dark red line) experienced 4°C temperature excursions from the nearby reef slope (measured at the 7.9 m depth mooring, Fig. 2 light green line) that coincided with day time low tides starting on March 14th, 2011 (Fig. 2 Time Point A). This temperature excursion on the reef flat is the result of the ebbing tide dropping below the reef crest, severing the water on the reef crest from that on the reef slope. This appeared to occur at a tidal height of approximately 1.1 m based on temperature data alone (Fig. 2). Further evidence of a clear separation of reef flat and adjacent waters can be seen in the depth record; at point B in Figure 2, the water depth (at 1.1 m depth mooring, green line) no longer follows a clear sinusoidal pattern and starts showing a rapid change in tidal height. These rapid changes occurred due to water rushing across the reef crest; up until these rapid changes, tidal exchange was being impeded. Based on reef flat water depth data, these rapid changes occur at a tidal height of 0.81 m (Fig. 2, green line at Time Point B). Jell and Webb (2012)

identified that the Heron Island reef crest becomes exposed at 0.6 m. This is probably a conservative estimate for the dates in which our study was conducted, based on temperature and tidal height (Figure 2 Time Point C). Based on Jell and Webb's (2012) estimate, collected real-time environmental data, and our concordant personal observations of reef flat aerial exposure, it is clear that many coral colonies on the Heron Island reef flat were aerially exposed (e.g., Fig. 1 photograph) twice a day from March 18-22, 2011.

Rain events appear to be coincident with a variety of the tides that exposed the reef crest and flat corals. The first of these may have occurred on March 14th, four days before the collection of experimental *Acropora aspera* colonies, however, this storm occurred when the tide was not at its lowest and consequently some coral colonies might not have been aerially exposed. The most pronounced of these events occurred on March 20th (Fig. 2 lower right) when a rain event that exceeded 40 mm h^{-1} fully coincided with low tide. Shortly after this, we visually observed bleaching across the reef flat (Fig. 1 photograph). On some larger colonies, bleaching was limited to the tops and upper parts of colony sides, which would have experienced aerial exposure during low tides; the lower sides of these colonies retained darker pigmentation. Less than 12 hours following this rainfall event, we collected *A. millepora* corals for the Millepora experiment.

It is important to note that the reef flat temperatures reported here were not unusual for this zone of the reef, nor were the air temperatures anomalous for March at Heron Island (Fig. 2 dark red and orange lines, respectively). When 2011 temperature data are compared to values from 2008, when sensors were originally deployed, through 2015 (Fig. 2 lower left graph), it is evident that 2011 temperatures were well within the variation typically observed in March and much lower than temperatures observed during November through February at Heron Island. Further, this reef flat typically experiences temperatures in excess of 34°C for much of the year (Figure 2 lower left); the maximum temperature recorded prior to and during our study period (March 13-28th, 2011) was maximum of 30.9°C.

Aspera *Experiment - Setup and Design*

Five colonies of "apparently healthy" *Acropora aspera* (cream morph) were collected from the tidal flat off of Heron Island, Australia on March 18, 2011. Twenty-six coral fingers were removed from each *A. aspera* colony. Two fingers from each colony were placed in an enclosed tank and heated to 34°C, and subsequently used to generate viral inoculate for injection treatments. The remaining 24 fingers from each colony were used as experimental fragments and placed upright in two racks (N = 12 fragments/rack). These fragments were allowed to acclimate for 24 hours in ~50 L glass aquaria at 28°C.

Using a fully crossed experimental design, fingers of each of the five colonies were exposed to a thermal stress (~2°C above ambient) or an ambient temperature treatment (N = 12 fingers/colony/temperature treatment), as well as injection with 200 µL viral inoculate (VI), heat-killed viral inoculate (HK), or virus-free seawater inoculate (SI) (N = 6 fingers/colony/injection treatment). Six fingers of each colony were not injected; these served as negative injection controls. Injections of viral and control inoculates began at 9:19 a.m. on March 19, 2011. Coral racks were rotated 180° in their tanks every 24 hours to minimize tank effects.

Aspera *Experiment - Preparation of Viral and Control Inoculates*

Thermally stressed tissue was water-picked into a sterile Ziploc bag from two fingers of each *A. aspera* colony heated to 34°C. Water-picking was conducted using 50 mL of viral-free seawater per coral finger. The resultant tissue slurry was homogenized and 0.45 µm-Sterivex filtered (as necessary) to remove large pieces of coral tissue, and then a total of ~90 mL was 0.22 µm-Sterivex filtered. Virus-like particles (VLPs) were then concentrated from the Sterivex filtrate using 30K Amicon Ultra-15 centrifugal filters (Millipore, MA, USA). Each Amicon filter (N = 6) was loaded with 15 mL of filtrate and spun for 10 to 20 minutes at 3392 x g, yielding ~8 mL filtrate total (i.e., ~10x concentrate). Epifluorescent microscopy confirmed the presence of VLPs in 100 uL aliquots of 0.45 and 0.22 µm Sterivex filtrate, as well as in 10uL of Amicon filtrate. 4 mLs of Amicon filtrate was wrapped in foil and stored at 4°C; this material was used as viral inoculate (VI). HK was prepared by boiling 4 mLs of Amicon filtrate for 1 hour. This material was then wrapped in foil and stored at 4°C until inoculations. Virus-free seawater inoculate (SI) was prepared using tangential flow-filtration (TFF) with a 100 kDa filter. SI was checked using epifluorescent microscopy to confirm the absence of VLPs and then frozen until inoculations (Thurber et al. 2009).

Millepora *Experiment – Setup and Design*

Four colonies of "apparently healthy" *Acropora millepora* were collected from the tidal flat off of Heron Island, Australia on March 20, 2011. Low tides coinciding with the full moon had led to aerial exposure and subsequent bleaching of many coral colonies on the tidal flat prior to sampling. *A. millepora* colonies were allowed to acclimate for 48 hours in a large raceway tank. Coral colonies were then placed in ~50 L individual glass aquaria with flow-through seawater running ~3 L per minute for the remainder of the experiment. Fingers of each of the four colonies were selected for injection with viral inoculate (VI) or heat-killed viral inoculate (HK), or identified as negative controls (N = 3 fingers/colony/treatment). All coral fingers were left *in situ* on the colony until sample processing at the conclusion of the experiment. A methods control (injection with viral-free seawater inoculate, SI) was not performed in the Millepora Experiment because this treatment had no discernible effect in the Aspera Experiment. Injections of viral and control inoculates began at 3:33 p.m. on March 22, 2011.

Millepora *Experiment - Preparation of Viral and Control Inoculates*

VI and HK inoculates were generated from 4 L of seawater that was collected from the tidal flat at low tide on March 21, 2011. Collections were performed by aspirating water from the interstitial spaces of bleached acroporid corals using a 60 mL syringe. An additional 4 L of seawater was also collected from the interstitial spaces of unbleached, apparently healthy acroporid colonies for subsequent virome generation; this material was not used for injections. Aliquots (1000 µL and 500 µL) of the 'bleached seawater' (BS) and 'healthy seawater' (HS) were immediately fixed with paraformaldehyde (4% final concentration), and then photographed and quantified in terms of their VLP concentration using epifluorescence microscopy and 100x SYBR Gold stain. The seawater was then stored overnight at 4°C.

The following morning, the BS and HS samples were concentrated using tangential flow filtration (TFF) to 50 and 33 mLs, respectively, and then 0.22 µm-Sterivex filtered. Epifluorescent microscopy was used to confirm the presence of viral-like particles before and following Sterivex filtering (as described in Thurber et al. 2009). Aliquots of the sterivexed HS (32.5 mL) and BS (35 mL) were

then fixed with chloroform (4% final volume) for virome generation. Each Sterivex filter was preserved at -20°C. A 10 mL aliquot of the 0.22 µm-Sterivex filtered BS sample was wrapped in foil and stored at 4°C until being used as VI. HK was prepared by boiling 5 mLs of the filtered BS sample for 1 hour; this material was wrapped in foil and stored at 4°C until the injections were performed.

Methodological Considerations – Virome Generation

Although we verified that we had abundant and pure viral particle DNA in our tissue preparations, we had significant difficulty conducting the Nextera preparation step required for Illumina sequencing. This required that we amplify the DNA prior to sequencing, and may have biased results in favor of some small circular genome types (i.e., *Circoviridae*, *Inoviridae*, and *Microviridae* in Table S1). MDA amplification can skew both the relative abundances of some taxa (most notably small circular genomes) and the cross-genome representation of those taxa (Abulencia et al. 2006, Yilmaz et al. 2010). However, the most comprehensive studies of MDA bias in viromes show that there is strong congruence ($R^2 \geq 0.97$) between viral metagenome replicates that have undergone this amplification procedure (Marine et al. 2014). Nevertheless, given that ssDNA virus sequence similarities (e.g., *Circoviridae*, Fig. 3, purple bar) comprised <5% of the virome and that this study did not compare results across viromes, the use of MDA is unlikely to have a significant impact on our conclusions regarding the core coral virome in *Acropora aspera*.

Phylogenetic Analysis - Methods

Contigs that had best hits to phylogenetically informative genes and that were >500 bp were selected from a single control saline-injected *A. aspera* virome for phylogenetic analysis. Contigs were translated into protein sequences using ExPASy Translate, an online translation tool[1]. Additional protein sequences to be included in each phylogenetic reconstruction were initially identified using PSI-BLAST searches to the NCBI viral non-redundant protein database, selecting only subject sequences with query coverage ≥ 60% and e-values $\leq 10^{-12}$ and $\leq 10^{-15}$ (MutS- and DNA polymerase-like contigs, respectively). Sequences were aligned as codons in MUSCLE using the default parameters. Alignments were examined by eye, curated in Gblocks to select phylogenetically informative areas of the alignment, and then block selection results were also examined by eye. Trees were constructed using the LG model in PhyML. The robustness of each phylogenetic tree was assessed using 100 bootstrap replicates. The threshold of bootstrap support used to collapse polytomies for the trees was 50/100. The online tool Phylogeny.fr was used to conduct the steps described above (Dereeper et al. 2008).

DNA Polymerase Tree

Many sequences in NCBI's viral protein database met the original threshold (e-value $\leq 10^{-12}$ and coverage ≥ 60%) for inclusion in a phylogenetic reconstruction of this 187 letter DNA polymerase-like amino acid sequence (translated from the original 497 bp contig in Table 1, Fig. S2, ENA Accession # LT009378). The threshold was thus made more stringent, with an e-value cutoff of ≤ 10⁻

[1] web.expasy.org/translate

[15], and appropriate sequences with a wide diversity of target hosts were selected for phylogenetic analysis. All homologous sequences are known gammaherpesviruses within the *Herpesviridae*.

MutS Tree

Five sequences in NCBI's viral protein database met the threshold (e-value $\leq 10^{-12}$ and coverage $\geq$ 60%) for inclusion in a phylogenetic reconstruction of this 184 letter MutS-like amino acid sequence (translated from the original 635 bp contig in Table 1, Fig. S3, ENA Accession # LT009379). All homologous sequences are known members of the candidate family *Megaviridae*, except for the Organic Lake phycodnavirus 1 sequence (GenBank Accession # ADX05918).

2. Supplementary Tables

Supplementary Table 1a. Ratios of contigs (C) and reads (R) with similarities to different viral Families based on analyses of best or top five tBLASTx hits. To avoid issues with results of zero, a one was added to every cell. Thus, values of 1 represent analyses that resulted in 0 similarities to a given viral Family. Boxed viral Families represent those that were differentially abundant across the analyses as measured by a deviation from the mean ratio.

Viral Family	Contigs Best	Contigs Top 5	Reads Best	Reads Top 5	R:C Best	R:C Top 5	R Top 5:R Best	C Top 5:C Best
Alloherpesviridae	3	31	17	526	5.67	16.97	**30.94**	**10.33**
Arenaviridae	1	3	1	100	1.00	**33.33**	**100.00**	3.00
Ascoviridae	2	4	4	11	2.00	2.75	2.75	2.00
Asfarviridae	1	3	3	6	3.00	2.00	2.00	3.00
Baculoviridae	11	70	105	910	9.55	13.00	8.67	**6.36**
Bicaudaviridae	1	1	2	2	2.00	2.00	1.00	1.00
Bunyaviridae	1	3	5	16	5.00	5.33	3.20	3.00
Caulimoviridae	13	56	85	394	6.54	7.04	4.64	4.31
Circoviridae	30	103	1099	4520	**36.63**	**43.88**	4.11	3.43
Closteroviridae	1	3	1	10	1.00	3.33	10.00	3.00
Fuselloviridae	2	2	1	1	0.50	0.50	1.00	1.00
Herpesviridae	73	130	817	1166	11.19	8.97	1.43	1.78
Hytrosaviridae	1	1	1	2	1.00	2.00	2.00	1.00
Inoviridae	15	16	670	672	**44.67**	**42.00**	1.00	1.07
Iridoviridae	6	9	107	121	17.83	13.44	1.13	1.50
Lipothrixviridae	1	1	3	3	3.00	3.00	1.00	1.00
Marseillevirus	3	7	7	52	2.33	7.43	7.43	2.33
Microviridae	1	1	23	60	**23.00**	**60.00**	2.61	1.00
Mimiviridae	18	98	174	723	9.67	7.38	4.16	**5.44**
Myoviridae	216	485	1062	2737	4.92	5.64	2.58	2.25
Nimaviridae	1	1	2	2	2.00	2.00	1.00	1.00
Papillomaviridae	1	1	6	47	6.00	**47.00**	7.83	1.00
Phycodnaviridae	42	80	389	757	9.26	9.46	1.95	1.90
Podoviridae	49	123	278	601	5.67	4.89	2.16	2.51
Polydnaviridae	6	106	85	1526	14.17	14.40	17.95	**17.67**
Poxviridae	14	28	58	174	4.14	6.21	3.00	2.00
Retroviridae	113	367	1258	4609	11.13	12.56	3.66	3.25
Siphoviridae	242	689	1860	6817	7.69	9.89	3.67	2.85
Tectiviridae	1	1	2	2	2.00	2.00	1.00	1.00
Togaviridae	2	2	2	2	1.00	1.00	1.00	1.00
Tymoviridae	1	1	1	2	1.00	2.00	2.00	1.00
Mean Ratio ± Stdev					8.21 ± 10.21	12.63 ± 15.57	7.64 ± 18.18	3 ± 3.37

Supplementary Table 1b. Concordance (R^2) values among different analysis approaches (best hits, top five hits) for contigs and reads generated from a single control, saline-injected *Acropora aspera* virome.

	Contigs Best	Contigs Top 5	Reads Best	Reads Top 5
**Contigs Best**		0.95	0.75	0.6
**Contigs Top 5**			0.75	0.75
**Reads Best**				0.88

3. Supplementary Figures

Supplementary Figure 1. Representative example of an individual control, saline-injected *Acropora aspera* fragment at the initiation (A) and end (B) of the Aspera experiment.

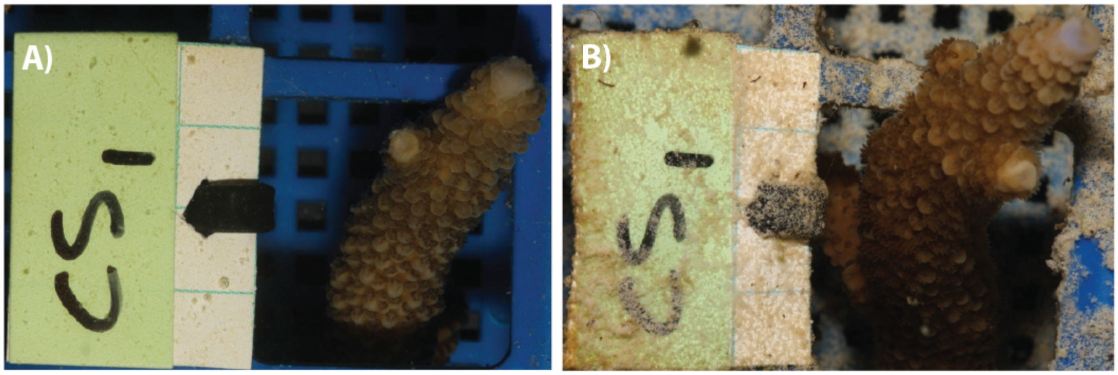

Supplementary Figure 2. Unrooted Maximum Likelihood reconstruction of the DNA polymerase gene. The *Acropora aspera* virome DNA polymerase sequence is deposited in the European Nucleotide Archive as Accession # LT009378.

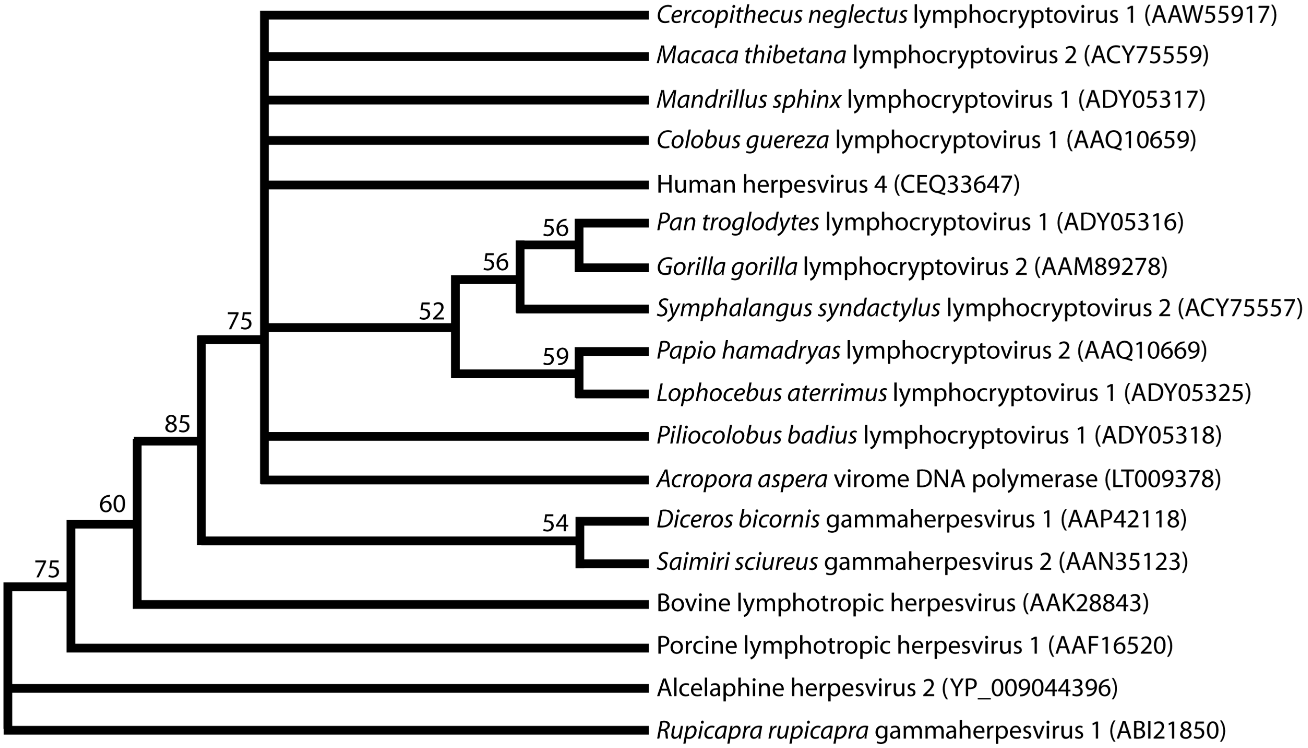

Supplementary Figure 3. Unrooted Maximum Likelihood reconstruction of the MutS gene. The *Acropora aspera* virome MutS sequence is deposited in the European Nucleotide Archive as Accession # LT009379.

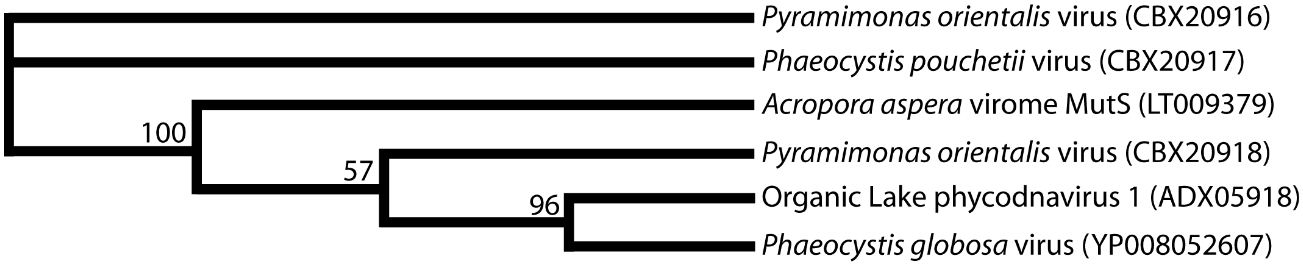

4. References

Abulencia, C.B., Wyborski, D.L, Garcia, J.A., Podar, M., Chen, W., Chang, S.H., Chang, H.W., Watson, D., Brodie, E.L., Hazen, T.C., and Keller, M. (2006). Environmental whole-genome amplification to access microbial populations in contaminated sediments. *Appl. Environ. Microbiol.* 72, 3291-3301.

Dereeper, A., Guignon, V., Blanc, G., Audic, S., Buffet, S., Chevenet, F., Dufayard, J.F., Guindon, S., Lefort, V., Lescot, M., Claverie, J.M., Gascuel, O. (2008) Phylogeny.fr: robust phylogenetic analysis for the non-specialist. *Nucleic Acids Res.* 36, doi: 10.1093/nar/gkn180.

Jell, J.S. and Webb, G.E. (2012) Geology of Heron Island and adjacent reefs, Great Barrier Reef, Australia. *Episodes*, 35: 110-119.

Marine, R., McCarren, C., Vorrasane, V., Nasko, D., Crowgey, E., Polson, S.W., and Wommack, K.E. (2014). Caught in the middle with multiple displacement amplification: the myth of pooling for avoiding multiple displacement amplification bias in a metagenome. *Microbiome* 2, 2–8.

Thurber, R.L.V., Haynes, M., Breitbart, M., Wegley, L., Rohwer, F. (2009) Laboratory procedures to generate viral metagenomes. *Nat. Protoc.* 4, 470-483.

Yilmaz, S., Allgaier, M., and Hugenholtz, P. (2010). Multiple displacement amplification compromises quantitative analysis of metagenomes. *Nature Methods* 7, 943-944.
